# A risk model based on autophagy-related lncRNAs for predicting prognosis and efficacy of immunotherapy and chemotherapy in gastric cancer patients

**DOI:** 10.18632/aging.203765

**Published:** 2021-12-12

**Authors:** Lei Gao, Juan Xue, Xiaomin Liu, Lei Cao, Ruifang Wang, Liangliang Lei

**Affiliations:** 1 Department of Gastroenterology, The First Affiliated Hospital, And College of Clinical Medicine of Henan University of Science and Technology, Luoyang, China; 2 Department of Clinical Laboratory, The First Affiliated Hospital, And College of Clinical Medicine of Henan University of Science and Technology, Luoyang, China; 3 Department of Gastrointestinal Surgery, The First Affiliated Hospital, And College of Clinical Medicine of Henan University of Science and Technology, Luoyang, China

**Keywords:** gastric cancer, autophagy, long non-coding RNA, prognosis, immunotherapy

## Abstract

Long non-coding RNAs (lncRNAs) are a class of non-protein-coding RNAs essential to the occurrence and development of gastric cancer (GC). We aimed to identify critical lncRNA pairs to construct a prognostic model and assess its performances in prognosis and efficacy prediction in GC patients receiving immunotherapy and chemotherapy. We searched transcriptome and clinical data of GC patients from The Cancer Genome Atlas (TCGA) database. Autophagy-related lncRNAs were identified using co-expression network analysis, and lncRNA pairs with prognostic value were selected using pairwise transcriptome analysis. The gene pairs were subjected to LASSO algorithm for identification of optimal gene pairs for risk model construction. Patients were classified into the low-risk and high-risk groups with the RiskScore as a cutoff. Finally, 9 optimal gene pairs were identified in the LASSO algorithm model for construction of a lncRNA prognostic risk model. For predictive performances, it successfully predicted a shorter survival of high-risk patients than that obtained in low-risk individuals (*P <* 0.001). It showed moderate AUC (area under the curve) values for 1-, 2-, and 3-year overall survival prediction of 0.713 and could serve as an independent predictor for GC prognosis. Compared to the low-risk group, high-risk patients had higher expressions of marker genes for immune checkpoint inhibitors (ICIs) and showed higher sensitivity to the chemotherapy agents, rapamycin, bexarotene, and bicalutamide. Our findings demonstrate a robust prognostic model based on nine autophagy-related lncRNA pairs for GC. It acts as an independent predictor for survival and efficacy prediction of immunotherapy and chemotherapy in GC patients.

## INTRODUCTION

GC is a common gastrointestinal malignancy originating from gastric mucosa epithelial cells, and it ranks fifth for cancer incidence and third for cancer deaths worldwide [1, 2]. The 2015 cancer statistics showed that GC has the third-highest cancer morbidity and the second-highest cancer mortality in China [3], ranking higher than most developed countries in Europe and North America [4]. However, early GC patients tend to be asymptomatic and cannot be diagnosed until the advanced stage, who have to face a risk of rapid metastasis and poor prognosis [2]. In the Chinese population, patients with stage III GC make up 50%-60% of the total GC cases, a higher prevalence than that achieved in South Korea or Japan. The 5-year survival rate of Chinese patients is only 35.9%, significantly lower compared to 60%-70% in South Korea and Japan. Current management for advanced GC in China mainly incorporates palliative surgery, radiotherapy, chemotherapy, biological therapy (or immunotherapy), and traditional Chinese medicine. Immunotherapy has been proven to benefit advanced GC patients with no response to chemotherapy [5]. As growing clinical trials center on personalized treatment for GC patients, individualized prescription of chemotherapy or immunotherapy has been another challenge in China.

Autophagy is a highly conservative process that maintains the stability of the intracellular environment by degrading the organelles damaged by aging and their misfolded proteins and reusing the products [6]. It is essential in various pathophysiological or metabolic processes in immunity, aging, tumors, nervous system diseases [7], and the occurrence and development of several cancer types (e.g., GC). For example, studies of Beclin-1 protein expression (a key inhibitor/activator of autophagy) analyzed 60 GC tissues and demonstrated that Beclin-1 was expressed in 83% of GC tissues, but it was sparsely expressed in normal gastric mucosa cells [8, 9]. Autophagy upregulates PD-L1 expression, the most important immune checkpoint inhibitor gene in GC, via the p62/SQSTM1-NF-*κ*B pathway [10]. Besides, kallikrein-related peptidase 6 (KLK6), a biomarker of GC associated with poor prognosis, could induce chemotherapeutic resistance by attenuating auranofin-induced cell death via an activation of autophagy in GC [11]. Consequently, identification of key regulators associated with autophagy can offer more precise diagnosis and personalized treatment for GC patients.

LncRNAs are a class of RNA transcripts with a length greater than 200nt. They are abundant and account for about 90% of the entire human transcriptome [12, 13] and regulate gene expression at the epigenetic, transcriptional, or post-transcriptional level but do not encode proteins [14]. LncRNAs have been shown to promote or inhibit autophagy via various pathways to determine carcinogenesis or carcinoma control. *LncRNA-HAGLROS* overexpression promotes the occurrence and development of GC via mTOR signal-mediated autophagy inhibition [15]. *LncRNA-SNHG11* fuels GC progression by activating the Wnt/*β*-Catenin pathway and carcinogenic autophagy [16]. *LncRNA-MALAT1* enhances autophagy-related chemical resistance by regulating the autophagy-related gene axis (ATG5 axis) in GC [17]. Alterations in the tumor immune microenvironment (TIME), particularly immune cell infiltration mediated by lncRNAs, are critical to patient prognosis [18, 19]. Autophagy-related lncRNAs have shown the potential to discriminate high-risk cancer patients from low-risk ones. Compared to a monogenic model, a multigenic model offers a more accurate prediction for cancer prognosis. For example, a prognostic risk model based on autophagy-related lncRNAs has been shown to exhibit high efficacy in predicting the prognosis of breast cancer [20] and bladder urothelial carcinoma [21]. However, models for prognosis prediction or assessment of immune cell infiltration and immune checkpoint gene expressions in GC have not been reported elsewhere, and if any, the feasibility of such models needs validation. In this study, we aimed to utilize the LASSO algorithm (COX regression analysis), pairwise transcriptome analysis, and iteration to identify optimal autophagy-related lncRNA pairs associated with GC prognosis for risk model construction. Differences in immune cell infiltration and sensitivity of patients to immunotherapy and chemotherapy between low- and high-risk groups were compared. This new tool for GC prognosis and treatment will shed light on the roles of autophagy-related lncRNAs in the TIME of GC.

## RESULTS

### Differentially expressed autophagy-related lncRNAs in GC

**Figure 1:**
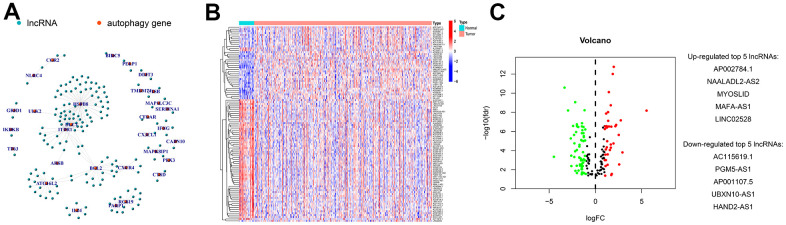
**Screening and identification of differentially expressed autophagy-related lncRNAs in GC.** (**A**) Construction of autophagy-related mRNA-lncRNA interactions using co-expression network analysis. Genes differentially expressed between GC and normal tissues were identified using differential analysis, and their expressions were visualized in (**B**) heatmaps and (**C**) volcanic plots. The identified top 5 increased or decreased autophagy-related lncRNAs in GC are also listed in panel C.

The transcriptome analysis of 375 GC and 32 normal tissues from TCGA identified 157 autophagy-related lncRNAs. The co-expression network revealed autophagy-related mRNA-lncRNA interactions in GC (Figure 1A). Among others, 102 autophagy-related lncRNAs differentially expressed between GC and normal tissues were confirmed, including 65 down-regulated lncRNAs and 37 up-regulated ones (Figure 1B, 1C).

### Construction of a GC risk model based on autophagy-related lncRNA pairs

**Figure 2:**
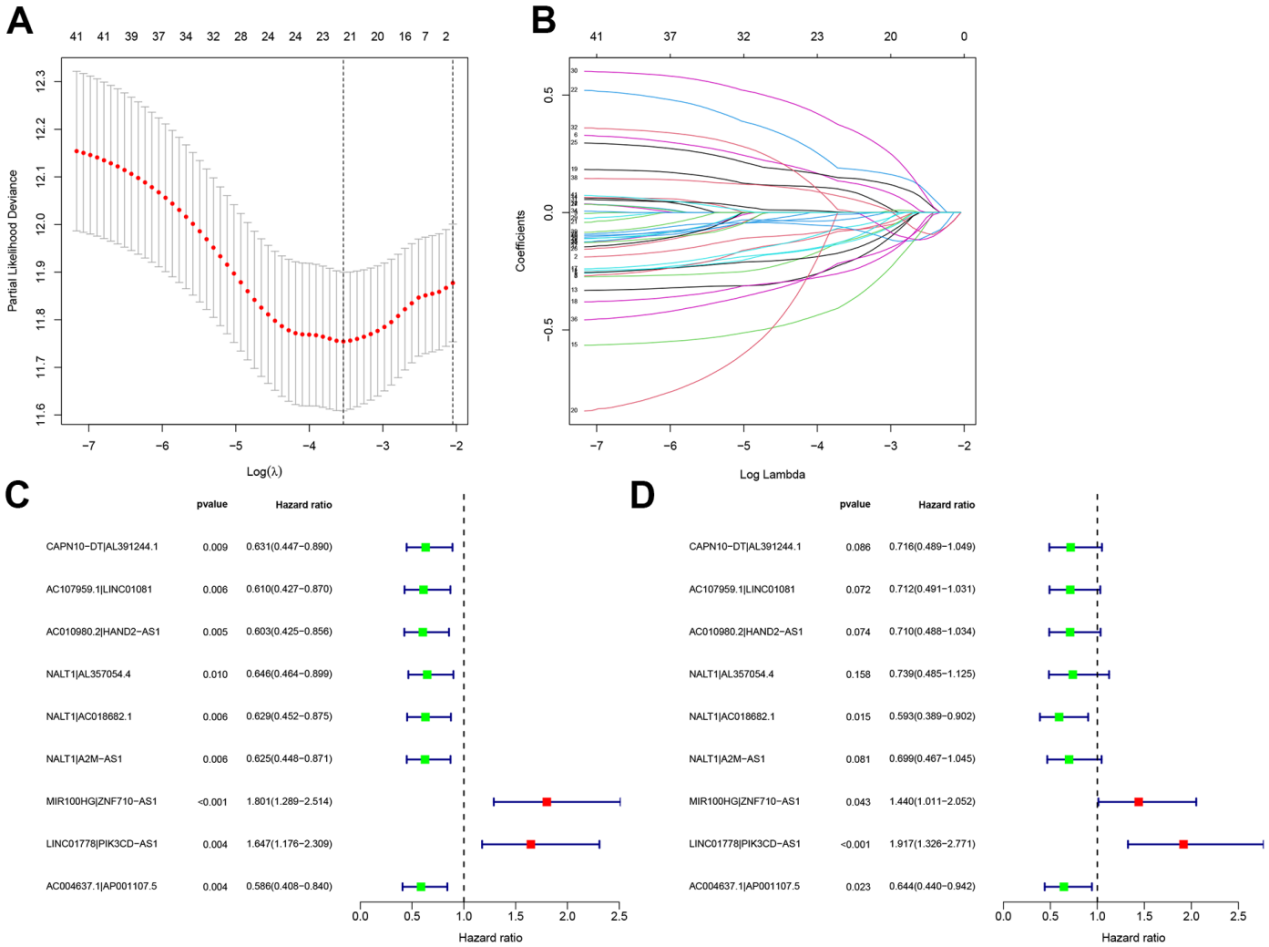
**Construction of a GC risk model based on autophagy-related lncRNA pairs.** (**A**, **B**) Univariate Cox regression and LASSO algorithm were utilized to identify optimal autophagy-related lncRNA pairs associated with GC survival. The optimal gene pairs were subjected to the Cox proportional hazard model using (**C**) univariate and (**D**) multivariate analyses. A prognostic model was constructed using a stepwise regression method. Hazard ratios were visualized in forest plots.

**Figure 3:**
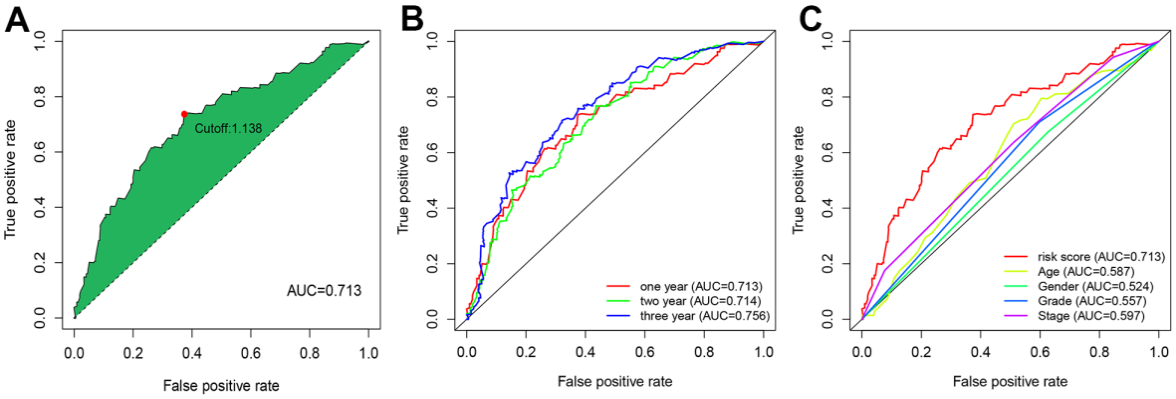
**ROC curve analysis revealed moderate accuracy of the risk model in prognosis prediction.** (**A**) Accuracy of the risk model in predicting the overall survival of GC patients. (**B**) Accuracy of the risk model in predicting 1-, 2-, and 3-year overall survival of GC patients. (**C**) Comparison of the prediction accuracy of the risk model with clinicopathological features, such as age, sex, and pathological grading.

We performed the pairwise transcriptome analysis of the 102 differential genes and found 2,895 autophagy-related lncRNA pairs showed significant differential expression in GC versus normal tissues. These genes were input to the LASSO algorithm, and nine optimal gene pairs associated with GC prognosis were ultimately confirmed using univariate Cox regression (Figure 2A, 2B) and visualized in forest plots of hazard ratios (Figure 2C, 2D). The median RiskScore for risk stratification was 1.138 (Figure 3A), upon which GC patients from TCGA were classified into the low- (a RiskScore < 1.138) and high-risk groups (a RiskScore > 1.138). For the accuracy of this model, the ROC curve analysis showed a moderate AUC for 1-, 2-, and 3-year overall survival prediction of GC patients of 0.713 (Figure 3B), more accurate than other clinicopathological features, such as age (AUC = 0.587), sex (AUC = 0.524), pathological grade (AUC = 0.557), and clinical stage (AUC = 0.597) (Figure 3C).

### Internal validation of the risk model for GC prognosis

**Figure 4:**
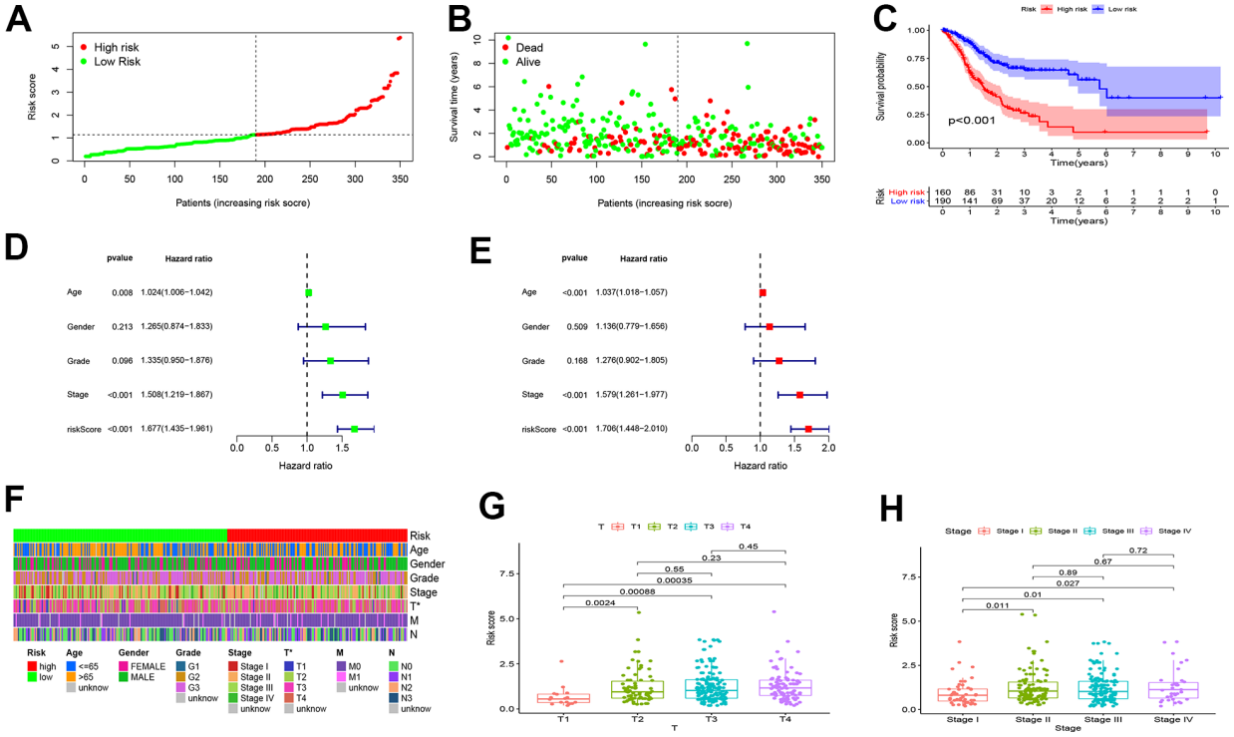
**Internal validation of the risk model for GC prognosis.** (**A**, **B**) The RiskScore and overall survival rate of each patient in the low- and high-risk group. (**C**) High-risk patients had shorter overall survival than low-risk patients. (**D**) Univariate and (**E**) multivariate Cox risk ratio analysis revealed that the risk model could predict GC prognosis independently. (**F**–**H**) The Wilcoxon signed-rank test showed that patients with high RiskScore often yielded late clinical stages, and the results were visualized in heatmaps and boxplots.

A TCGA cohort incorporating 160 high-risk patients and 190 low-risk patients was used for internal validation of the predictive efficacy of the risk model. As shown in Figure 4A, 4B, the risk score was negatively correlated with the overall survival rate of GC patients. In Kaplan-Meier survival analysis, high-risk patients had shorter overall survival than low-risk patients (*P* < 0.001) (Figure 4C). Univariate (HR=1. 677, 95%CI: 1.435-1.961, *P* < 0.001) and multivariate Cox risk ratio analysis (HR=1.706, 95%CI: 1.448-2.010, *P* < 0.001) showed that the risk model could serve as an independent prognostic predictor for GC (Figure 4D, 4E). The RiskScore also had significant correlations with T stage (Figure 4G) and clinical staging (Figure 4H) that patients with late clinical stages often yielded higher RiskScores. The correlations were visualized in heatmaps and boxplots (Figure 4F).

### Differences in tumor-infiltrating immune cell (TIC) landscape between low- and high-risk patients with GC

**Figure 5:**
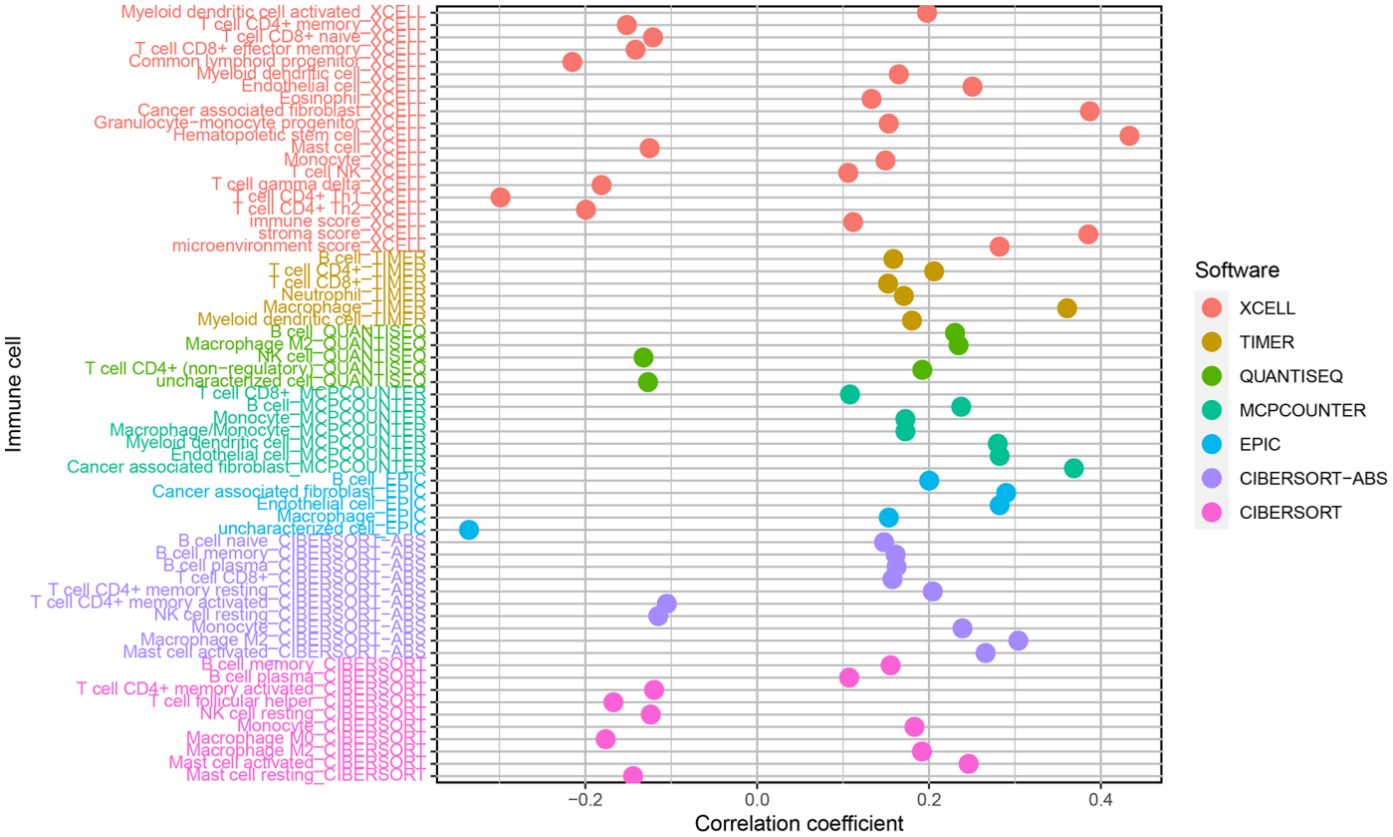
Correlations of the risk model (RiskScore) based on nine autophagy-related lncRNA pairs with tumor-infiltrating immune cell landscape.

**Figure 6:**
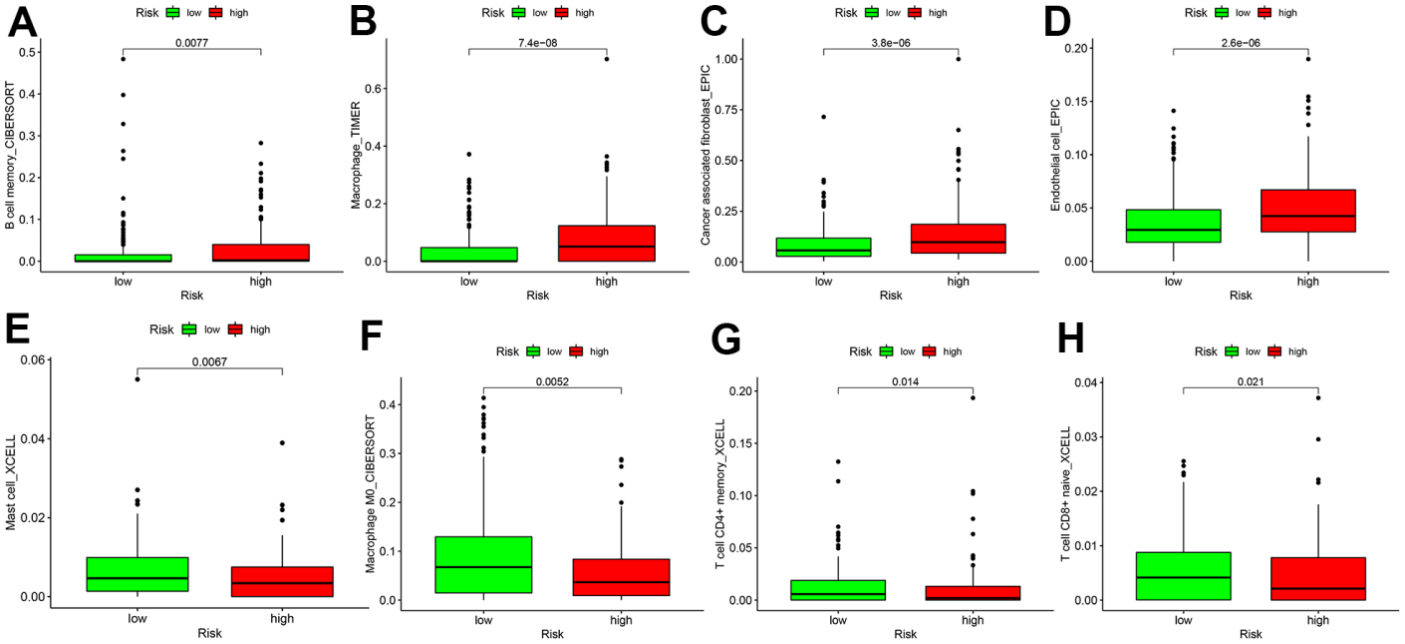
**Differences in tumor-infiltrating immune cell subpopulations between GC low- and high-risk patients.** (**A**) B cell memory; (**B**) Macrophage cell; (**C**) Cancer associated fibroblast cell; (**D**) Endothelial cell; (**E**) Mast cell; (**F**) Macrophage M0 cell; (**G**) CD4^+^ T memory cell; (**H**) T cell CD8^+^naive cell.

As TICs determine cancer cell fate and involve in GC prognosis, we assessed whether the nine autophagy-related lncRNA pairs in the risk model (RiskScore) are associated with the TIC landscape supporting tumor progression using the Wilcoxon signed-rank test (Figure 5). High-risk patients exhibited a higher degree of B memory cell, cancer-associated fibroblast (CAF), endothelial cell, macrophage infiltration versus low-risk patients (Figure 6A–6D). An increased degree of M0 macrophage, activated and memory CD4^+^ T cell, naive CD8^+^ T cell, and mast cell infiltration was present in the TIME of low-risk patients (Figure 6E–6H).

### Sensitivity of GC patients to chemotherapy agents and ICIs

**Figure 7:**
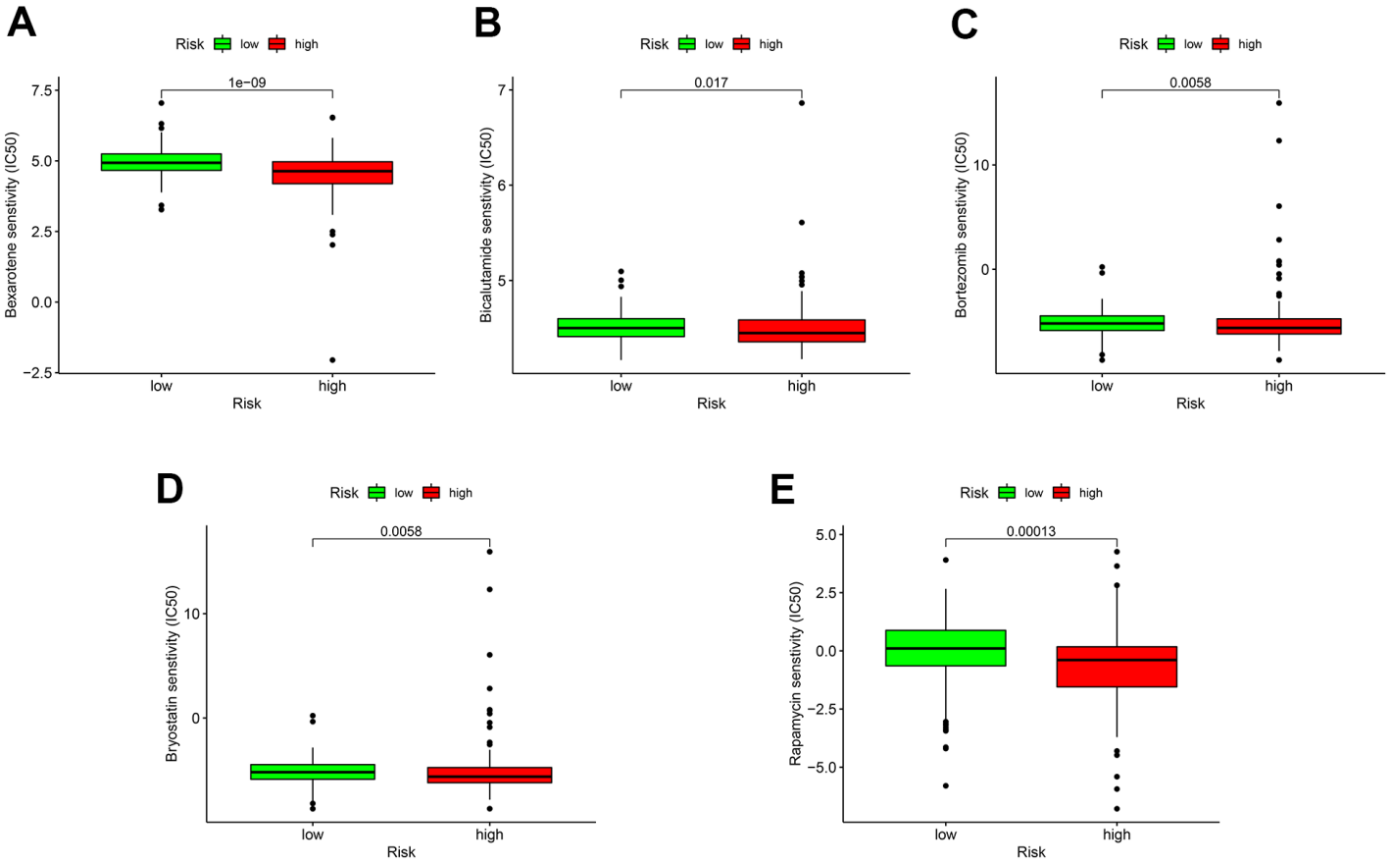
**Sensitivity of low- or high-risk patients to five common chemotherapy agents.** The *y*-axis represents 50% inhibitory concentration (IC_50_). (**A**) Bexarotene; (**B**) Bicalutamide; (**C**) Bortezomib; (**D**) Bryostatin; (**E**) Rapamycin.

**Figure 8:**
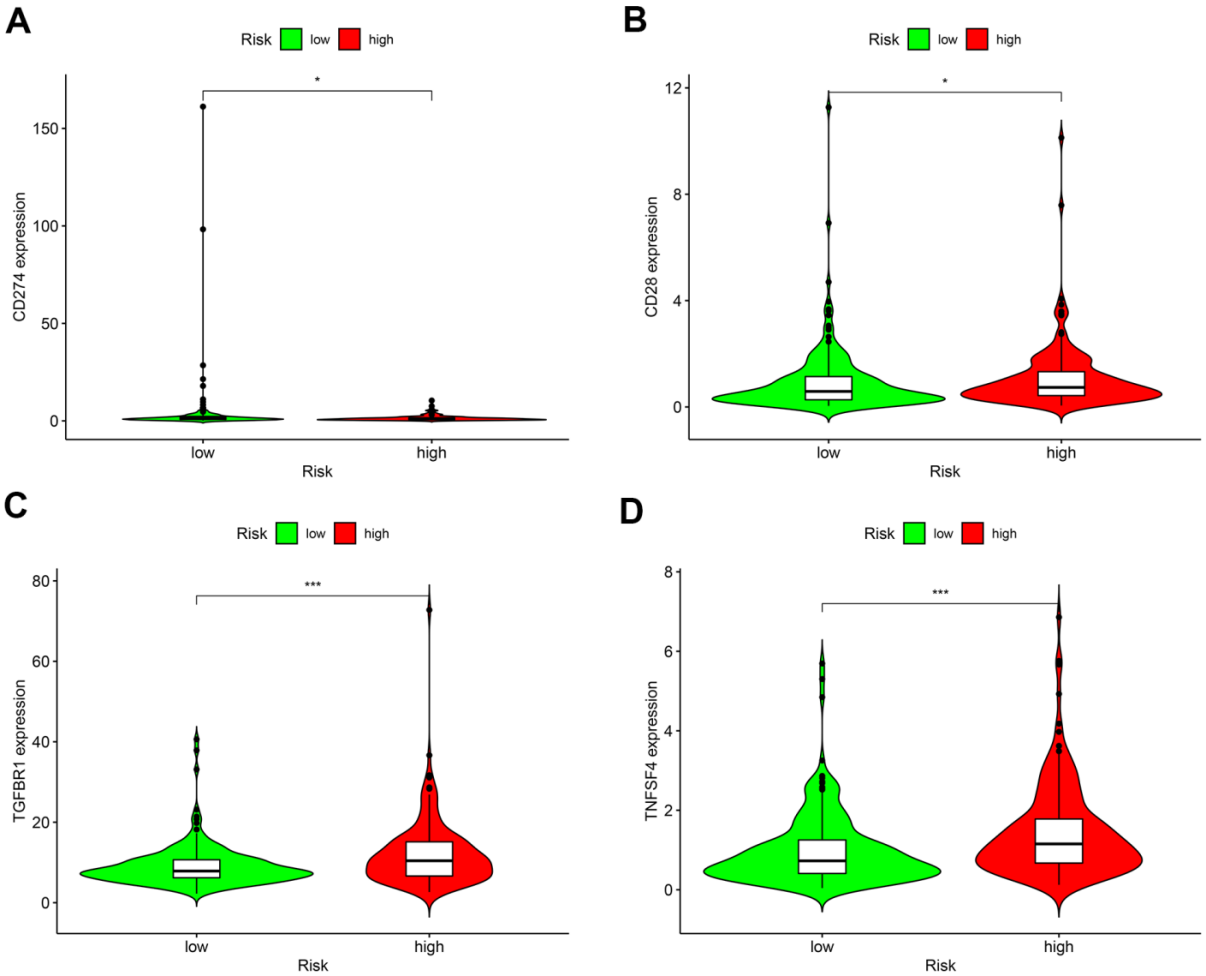
**Differences in the expressions of marker genes for immune checkpoint inhibitors between low- and high-risk patients.** (**A**) *CD274 (PD-L1)*; (**B**) *CD28*; (**C**) *TGFBR1*; (**D**) *TNFSF4* (*OX40L*). *P < 0.05, **P < 0.01, ***P < 0.001.

As for chemotherapy sensitivity, the five common agents mentioned above were selected for comparisons of IC_50_ values between the low- and high-risk groups. GC high-risk patients showed lower IC_50_ values for bexarotene, bicalutamide, bortezomib, bryostatin, and rapamycin (all *P* < 0.05) (Figure 7), indicating that patients in this group may have high sensitivity to these chemotherapy agents. The correlation of low- or high-risk GC patients with ICI marker genes was also assessed, and the results showed that *CD274* (*PD-L1*), *CD28*, *TGFBR1*, and *TNFSF4* (*OX40L*) expressions were all up-regulated in high-risk GC patients compared with the low-risk ones. All differentially expressed ICI marker genes were detailed in Figure 8. These results suggest that the RiskScore model based on autophagy-related lncRNA pairs shows immunotherapy and chemotherapy benefits to GC patients.

## DISCUSSION

GC is one of the most frequent malignancies worldwide, with morbidity and mortality still mounting [22]. Although the 5-year survival rate of early GC patients can reach more than 95%, the majority of patients are diagnosed at the late stage, with an unfavorable prognosis and insurmountable drug resistance [22, 23]. Currently, insufficient biomarkers have been documented for outcome prediction for GC patients after treatment [24, 25]. Autophagy has been shown to participate in the occurrence and development of GC, and autophagy-related biomarkers may aid in more accurate diagnosis early on [26]. In the last decade, autophagy-relate lncRNA signature-based cancer markers have been studied and proven to show tight associations with cancer cell growth and survival, chromatin modification, genome imprinting, and other significant biological functions [27]. For example, upregulation of *lncRNA-SNHG11* in GC correlated with dismal patient outcomes, and aggravated oncogenic autophagy to facilitate cell proliferation and metastasis via triggering the Wnt/β-Catenin pathway and oncogenic autophagy. Moreover, *lncRNA-MALAT1* functioned as a competing endogenous RNA (ceRNA) for *miR-23b-3p* and weakens the prohibitive effect of *miR-23b-3p* on ATG12, resulting in chemo-induced autophagy and chemoresistance in GC cells [28]. Therefore, it is imperative to identify autophagy-specific lncRNAs associated with GC survival and clinical treatments. In the present study, we constructed a risk model (RiskScore) based on autophagy-related prognostic lncRNA pairs and demonstrated a good performance of this signature in prognosis prediction and assessment of the sensitivity of GC patients to ICIs and common chemotherapy agents.

Pairwise transcriptome analysis is commonly utilized to screen survival-associated RNAs in cancers, which is effective for fast cancer gene marker identification for prognosis prediction. In the present work, we obtained 102 autophagy-related lncRNAs differentially expressed in GC via differential analysis of the transcriptome data of GC tissues, which were subsequently subjected to pairwise transcriptome analysis [29]. After 2,895 effective autophagy-related lncRNA pairs were screened and samples with missing or incomplete information were discarded, they were merged with survival data from TCGA with the limma package. Among these gene pairs, 42 prognostic gene pairs were selected using univariate Cox regression (*P* = 0.01) and were input to the LASSO regression model for cross-validation. Ultimately, 9 optimal gene pairs were obtained for risk model construction. In the above procedures, the gene pairs selected via pairwise transcriptome analysis are exempt from batch adjustments--required when transcriptome data from TCGA for model construction are merged with chips or PCR data from clinical studies--and repetitive adjustments for the risk model. Instead, this method increases the feasibility of the risk model in clinical application. The modified LASSO algorithm we used in our analysis was proposed by Sveen et al. Absolute gene expression can reflect differentially expressed genes between cancer and normal samples [30]. This modeling method based on the rank of occurrence frequency of differentially expressed genes is effective in assessing the performance of lncRNA pairs prognosis prediction [30], which showed moderate AUC values for 1-, 2-, and 3-year survival prediction. This result was further supported by Kaplan-Meier survival analysis. The optimal RiskScore cut-off was calculated for risk stratification, and comparisons of the risk model (RiskScore) based on the nine autophagy-related lncRNA pairs with other clinical indicators revealed the better performance of the risk model than that of age, sex, pathological grading, and clinical staging.

For far too long, tumors with ubiquitous mutations have been repeatedly detected. Tumor cells bearing mutations can produce abundant new antigens that are subsequently recognized by T cells, stimulating the selective recruitment of immune cells [31]. So tumor stroma is infiltrated with immune cells, which is a critical mechanism for immunotherapy efficacy, notwithstanding the type, location, and density of immune cells [32]. We also explored whether a relationship was present between immune cell infiltration and the risk model (RiskScore) in high-risk patients. The results showed that autophagy-related lncRNAs were significantly correlated with tumor infiltration by CAFs, hematopoietic stem cells, CD4^+^ Th1, and other immune cells. In high-risk patients, the TIME consists of abundant B memory cells, cancer-associated fibroblasts (CAFs), endothelial cells, and macrophages, which supports tumor growth; whereas low-risk patients showed more M0 macrophages, activated and memory CD4^+^ T cells, naive CD8^+^ T cells, which have a strong effect on tumor cell killing. Therefore, the current risk model may offer efficacy prediction for immunotherapy. The degree of tumor infiltration by CD8 ^+^ T cells has been proven to predict the prognosis in some cancer types, such as melanoma, ovarian, and colon [33]. Increased naive and memory B cell, resting memory CD4^+^ T cell, follicular helper T (Tfh) cell, monocyte, resting natural killing (NK) cell, M0 and M1 macrophage, resting mast cell, and activated mast cell infiltration suggests significant immune regulation in the cancer microenvironment, which offers more options for immunotherapy and more targets for sensitivity assessment [34–36].

Radiotherapy and chemotherapy are mainstream treatments for GC patients following surgical resection [37]. As predicted by the risk model, high-risk patients showed lower IC_50_ values for bexarotene, bicalutamide, bortezomib, bryostatin, and rapamycin. This finding shows the potential of this model as a guide to personalized prescriptions of chemotherapy agents for GC patients. High heterogeneity of GC patients and complex tumor-host differences lead to a high mutation rate in cancer cells. Patients bearing these mutations tend to develop chemotherapy resistance and ultimately turn to immunotherapy [38, 39]. In a healthy immune system, immune cells can recognize and eliminate cancer cells with mutations to reduce the likelihood of cancer cell proliferation. In the TIME, cancer cells escape from immune surveillance and proliferate rapidly, or called tumor escape [19]. Immunotherapy can restore immune response via blocking immune checkpoint receptors and their ligands, thus managing to reaccelerate immune-mediated destruction of tumor cells [19, 40]. The multicenter, phase 2 Keynote-059 study proved that the third-line pembrolizumab was effective in the treatment of advanced GC, in which the objective response rate was 60.0% for combination therapy and 25.8% for monotherapy [41]. The efficacy of nivolumab was ascertained by the ATTRACTION-2 trial, which extended the overall survival (12-month overall survival rates were 26.2% with nivolumab versus 10.9% with placebo) of advanced GC patients who had failed prior chemotherapy [42]. It has been well accepted that the ICI marker genes, PD-1 or CD274 (PD-L1), acts as important drug targets in cancer immunotherapy [43]. Other immune checkpoint-related genes *as CD28*, *TGFBR1*, and *TNFSF4 (OX40L)* have been frequently reported to engage in cancer immune regulation in ICI research [44–46]. Our findings demonstrated that *CD274* (*PD-L1*)*, CD28*, *TGFBR1*, and *TNFSF4* expressions expression were elevated in high-risk GC patients compared to low-risk controls. Therefore, this model can bring immunotherapeutic benefits to patients at higher risk of advanced GC.

Limitations of this study incorporate an insufficient sample size and a lack of validation using data from our center. For external validation, we initially included a validation cohort from GEO, which failed to fully cover the autophagy-related lncRNA pairs selected for risk model construction, so this cohort was removed. Our future study will center on clinical validations of this model in GC, which may provide strong results for its application in a timely manner.

## CONCLUSIONS

Our findings demonstrate a robust prognostic model based on nine autophagy-related lncRNA pairs for GC. It acts as an independent predictor for survival and efficacy prediction in GC patients receiving immunotherapy or chemotherapy. Our conclusion and the feasibility of the risk model require more accurate validation in future large-sample studies.

## MATERIALS AND METHODS

### Construction of an autophagy-related mRNA-lncRNA network and identification of autophagy-related lncRNA pairs

We searched gene-expression and clinical data of GC patients from The Cancer Genome Atlas (TCGA, https://gdc.cancer.gov/) and identified GC-related lncRNAs and mRNAs according to their Ensembl IDs (http://asia.ensembl.org). Autophagy-related lncRNAs were obtained using the co-expression network analysis and visualized in different nodes connected by lines representing autophagy-related mRNA-lncRNA interactions. Genes with the correlation coefficient > 0.6 and *P*-value < 0.001 were selected and subjected to pairwise transcriptome analysis for selection of autophagy-related lncRNA pairs, which could circumvent batch adjustments. Expressions of paired lncRNAs were ranked using a 0-or-1 matrix. It is recorded as 1 if the expression level of lncRNA-A is greater than that of lncRNA-B; otherwise, it is recorded as 0. LncRNA pairs with stable expression order, whether 0 or 1, in 20%-80% of all samples were selected as stable autophagy-related lncRNA pairs [29].

### Identification of autophagy-related prognostic lncRNA pairs and construction of a risk model

These gene pairs were input to the LASSO algorithm to screen out optimal lncRNA pairs associated with GC prognosis. This method prevents over-fitting during modeling [47]. A model based on the optimal lncRNA pairs was developed with Cox regression, and the median RiskScore was calculated (the sum of Cox regression coefficient multiplied by the expression value of each lncRNA) for risk stratification. Patients were stratified into low- and high-risk groups with the RiskScore as a cut-off. The accuracy of the model in 1-, 2-, and 3-year survival prediction was assessed using ROC curve analysis and determined by AUCs.

### Internal validation of the risk model

We compared survival differences between low- and high-risk groups using Kaplan-Meier survival analysis. Comparisons of prediction performances of the model with other clinicopathological prognostic indicators were performed using a multi-index ROC-based methodology. Their differences were determined using the Chi-square test, marked with asterisks, and visualized in heatmaps. The survival, survivalROC, ggpubr, and pHeatmap packages in R language were used [48].

### Tumor-infiltrating immune cell landscape between the two risk groups

We used TIMER (http://cistrome.dfci.harvard.edu/TIMER/), XCELL (https://xcell.ucsf.edu/), QUANTISEQ, MCPCOUNTER, EPIC, CIBERSORT-ABS, and CIBERSORT algorithms [29] to assess differences in immune cell subpopulations between low- and high-risk groups. Their differences were compared using the Wilcoxon signed-rank test.

### Drug sensitivity analysis of immune checkpoint inhibitors (ICIs) and chemotherapy agents in GC

We were very interested in characterizing the sensitivity to commonly used ICIs and chemotherapy agents in low- and high-risk GC patients. Common chemotherapy agents (i.e., bexarotene, bicalutamide, bortezomib, bryostatin, and rapamycin) were selected for analysis. Differences in 50% inhibitory concentration (IC_50_) were compared between the two risk groups using Wilcoxon signed-rank test, and the results were visualized with pRophetic and ggplot 2 in R. As several chemotherapy agents have been proven ineffective for advanced GC patients, they are being replaced by the use of immunotherapy agents, particularly the most promising ICIs. We compared differences in expression levels of ICI marker genes, and the results were visualized in violin plots using the ggpubr package in R.

### Statistical analyses

All statistical analyses were performed in software R (Version 4.0.2). The differential expression of autophagy-related lncRNAs was determined using the limma package in R at the false discovery rate (FDR) < 0.05 and log2 fold change (FC) > 2. Expression data of the autophagy-related lncRNA pairs and patient survival data were integrated using the limma package and subject to univariate Cox combined with LASSO regression analysis. Gene pairs with a *P* < 0.01 were selected. Prognostic factors associated with GC risk were identified using univariate and multivariate Cox regression analysis to determine whether the model could be considered an independent prognostic indicator for GC. The survival curves between low- and high-risk groups, predicted by the model were plotted using Kaplan-Meier survival analysis. Comparisons of clinicopathological features between the two risk groups were evaluated using the Wilcoxon signed-rank test, with the significance level set at *P*-value < 0.05.
